# The CD4^+^ T-lymphocyte count is an important predictor for the prognosis of cryptococcosis

**DOI:** 10.1007/s10096-016-2880-9

**Published:** 2016-12-29

**Authors:** Y. Ding, P. Li, Q. He, H. Wei, T. Wu, D. Xia, M. Tan, Y. Shi, X. Su

**Affiliations:** 1 Department of Respiratory and Critical Care Medicine, Jinling Hospital, Medical School of Nanjing University, Nanjing, China 210002; 2 Department of Respiratory and Critical Care Medicine, Jinling Hospital, Southern Medical University, Nanjing, China 210002; 3 Department of Infectious Disease, Nanjing Second Hospital, Nanjing, China 210002

## Abstract

There is great heterogeneity of immunity among patients with cryptococcosis, and severe immunodeficiency can lead to negative clinical outcomes. Underlying disease is a poor surrogate for immune status and inferior in predicting an individual’s prognosis. This study was intended to determine whether T-lymphocyte subgroups would be more suitable indicators regarding the severity of infection and clinical outcomes of such patients. We retrieved clinical data on 101 patients with cryptococcosis and compared the validity of multiple parameters (underlying disease and T-lymphocyte subgroups) in predicting the severity of infection and clinical outcome in these patients. For patients with CD4^+^ T-lymphocyte counts lower than 400/μL, the odds ratio of disseminated cryptococcosis was 23.3 (*P* = 0.005). There was a moderate negative correlation between CD4^+^ T-cell count and Apache II score (−0.609, *P* < 0.001). Mortality among patients with low levels of CD4^+^ T lymphocytes was significantly higher than among those with normal levels (23.8% vs 5.3%, *P* = 0.016). However, the difference was not significant if the patients were grouped by underlying disease (*P* = 0.067). The CD4^+^ T-lymphocyte count in peripheral blood is a simple and more accurate biomarker for predicting severity of infection and clinical outcome in patients with cryptococcosis.

**Electronic supplementary material:**

The online version of this article (doi:10.1007/s10096-016-2880-9) contains supplementary material, which is available to authorized users.

## Introduction

Cryptococcosis is a potentially life-threatening systemic mycosis [1]. Inhalation of *Cryptococcus* spores is the main route of infection; such spores may remain isolated in the lungs or spread to other organs, depending on the host’s immune status [2]. The central nervous system (CNS) is considered to be the most vulnerable organ involved in this process. Disseminated disease, especially CNS involvement, often points to a need for timely, aggressive treatment [3] and a much higher mortality without it [4]. Thus, the risk factors for dissemination (i.e., the host’s immune status or underlying diseases) are always the top concerns of clinicians seeking to manage this infection.

With their significantly heterogeneous levels of immunity, those susceptible to infection with *Cryptococcus* include patients with human immunodeficiency virus (HIV)/adult immunodeficiency syndrome (AIDS), organ transplant recipients [5], and patients with sarcoidosis [6], liver failure [7], leukemia [8], or diabetes mellitus [9] as well as those receiving aggressive drug therapy [10]. Cryptococcosis may, in fact, arise even in patients with no underlying disease [11, 12]. In other words, these patients may have various degrees of immune deficiency and risk for dissemination. Up to now, clinical guidelines have recommended that clinicians evaluate their patients’ underlying diseases and take the findings as one of the main classification criteria for planning a therapeutic regimen. But we found this approach inadequate. In short, it is imperative to find other parameters for evaluating patients’ immune status and predicting disease severity and clinical outcome.

Many studies suggest that cell-mediated immunity, especially in terms of T lymphocytes, plays an important role in countering cryptococcal infection [13–15]. For example, AIDS patients have been characterized by CD4^+^ T-cell depletion and inversion of the CD4^+^/CD8^+^ ratio [16]. One recent study from Germany suggested that routine screening for cryptococcosis by testing with cryptococcal antigen (CRAG) is highly recommended for AIDS patients with CD4^+^ T-cell counts ≤200/μL [17]. Another example involves the use of rabbit anti-thymocyte globulin and alemtuzumab, which have been given to organ transplant patients to counteract acute rejection; however, this treatment can induce a substantial decline in the number of CD4^+^ T cells and thus increase the risk of cryptococcal infection [18]. Idiopathic CD4^+^ T lymphocytopenia has also been reported to be an important predisposing factor for cryptococcosis [19, 20]. By the recruitment of granulocytes and macrophages in the lungs and the production of IL-12, TNF-α, and IFN-γ, CD4^+^ T cells have been shown to protect mice against cryptococcal infection [15]. All these examples show that T lymphocyte subgroups play an important role in the pathogenesis of cryptococcosis.

Clinicians need a simple, straightforward indicator to determine an individual’s immune status and thus to predict the risk of disseminated cryptococcal disease and its potential severity, also keeping in mind other possible underlying diseases. T-lymphocyte subgroups constitute quantitative parameters; their numbers can readily be determined in routine clinical practice. With this in mind, we launched a retrospective study to compare the predictive role of underlying diseases and T-lymphocyte subgroups for patients with cryptococcosis and to find out which parameter would have the greatest predictive power.

## Methods and materials

We retrieved the clinical data of adult cryptococcosis patients in Jingling Hospital, and the Second Hospital of Nanjing, Nanjing, China. The diagnosis of cryptococcosis was confirmed either by histopathological examination of tissue samples (obtained by percutaneous lung biopsy, surgical resection, bronchoscope biopsy) or by positive staining/culture results from cerebrospinal fluid and/or blood. Clinical diagnoses were made on the basis of typical clinical manifestations and the cryptococcal antigen test. We ruled out those suspected cases that did not fulfill our diagnostic criteria as well as cases that did not include data on the peripheral blood T-lymphocyte subgroups at the time of diagnosis.

According to the classification criteria of the clinical practice guidelines for the management of cryptococcal disease published by the Infectious Disease Society of America in 2010, patients with cryptococcosis fall into several groups: the AIDS group, organ transplantation recipient (OTR) group, and NHNT (non-HIV-infected and non-transplant host) group. Patients with HIV infection and those who have received solid organ transplants were allocated to the AIDS and OTR groups, respectively. Since the heterogeneity of the NHNT group is quite large, this group was divided again [21] into the NHNT-1 group (where the patient suffered from one or more diseases that can cause mild or moderate immune deficiency and increase the risk of infection, such as autoimmune disease, diabetes mellitus, hematological disease, and so on) and the NHNT-2 group (comprising patients with no detectable immunological defects or underlying diseases).

Flow cytometry performed by professional technicians was used to analyze T-lymphocyte subgroups among our patients at admission. In addition, the findings on chest CT were described by two radiologists at Jinling Hospital.

Apache II scores and acute physical and chronic health evaluations were widely applied to assess the severity of disease [12, 22]. Integral scores ranged from 0 to 71, a higher score indicating a more severe condition and poorer prognosis. This score was determined according to the patient’s condition prior to antifungal therapy. Survival at the conclusion of our study (follow-up of at least 3 months for each patient [4]) constituted the clinical outcome. All data were acquired from medical records or telephone follow-up.

### Statistical analysis

Data were described as means (standard deviation) and percentages. One-way analysis of variance, *t*-tests, and chi-square tests were used in comparing the baseline characteristics. Because of the heterogeneity of our data, T-lymphocyte subgroups were expressed in terms of the median; the Kruskal-Wallis H test was applied in comparing the four groups; subsequently the Nemenyi test was used to make paired comparisons. The cutoff value of parameters was determined by receiver operating characteristic (ROC) curves and Youden’s index. The association was assessed by Spearman’s correlation analysis. The chi-square test was used in comparing groups. Significance was defined as *P* <0.05 with two-sided analysis. We analyzed all data with SPSS software, version 17.0.

## Results

Between January 2009 and July 2015, a total of 106 proven cases of cryptococcosis were collected, including an AIDS group (n = 30), an OTR group (n = 15), a NHNT-1 (n = 32) group, and a NHNT-2 (n = 29) group. The AIDS patients were characterized by a substantial decrease in the number of CD4^+^ T cells, and OTR patients had to be treated with immunosuppressants, so their T-lymphocyte counts were also low. All T-lymphocyte counts were routinely evaluated in these patients. Two NHNT-1 cases and three NHNT-2 cases had not been studied for T-lymphocyte subgroups. In sum, 101 cases met all our criteria and were therefore included in this study. There were 73 male and 28 female patients from 16 to 78 (41.4 ± 13.6) years of age. Of the total, 26 patients had no comorbidity (NHNT-2 group). The other 75 patients were assigned to the AIDS group (n = 30), OTR group (n = 15), or NHNT-1 group (n = 30). The demographic data for each group are listed in Table 1.

**Table 1:** Demographic data on 101 patients with cryptococcosis, 2009–2015

Characteristic	AIDS, n = 30	OTR, n = 15	NHNT-1, n = 30	NHNT-2, n = 26	*P* value
Male	26(86.7%)	11(73.3%)	17(56.7%)	19 (73.1%)	0.074
Age (years)	37.8 ± 11.5	42.8 ± 12.9	42.3 ± 17.4	43.9 ± 10.4	0.219
Underlying disease					
SOT (kidney)	None	15	None	None	
Renal disease	None	15	12(40%)	None	
AID	None	None	11(36.7%)	None	
Hemopathy	None	None	5(16.7%)	None	
Diabetes mellitus	None	3(20%)	6(20%)	None	
ILC	None	None	4(13.3%)	None	
Liver dysfunction	None	None	1(3.3%)	None	
Site of infection					
Disseminated	26(86.7%)	12(80%)	15(50%)	3(11.5%)	<0.001
Local	4(13.3%)	3(20%)	15(50%)	23(88.5%)	

*AID* autoimmune disease, *AIDS* acquired immunodeficiency syndrome, *ICL* idiopathic CD4+ lymphopenia, *NHNT* non-HIV-infected and non-transplant host, *OTR* organ transplantation recipient, *SOT* solid organ transplant

Renal disease in the NHNT-1 group included lupus nephritis (n = 8), nephritic syndrome (n = 2), podocytopathy (n = 1) and allergic tubule-interstitial nephritis (n = 1).

Autoimmune disease included SLE (n = 8), pemphigoid (n = 1) and rheumatoid arthritis (n = 2).

Hemopathy included malignant lymphoma (n = 2), myelodysplastic syndromes (n = 2) and allergic purpura (n = 1).

The data was described in percentage or mean (standard deviation). One-way analysis of variance and chi-square test were used in comparison between groups.

All 15 patients in the OTR group were kidney transplant recipients. The NHNT-1 patients suffered from various underlying diseases, including lupus nephritis, nephritic syndrome, lymphoma, and so on. NHNT-2 patients had no detectable diseases that could compromise their immune systems. Except for the underlying diseases, the baseline characteristics of each group were comparable (*P* > 0.05) (Table 1).

The definition of disseminated cryptococcosis includes the involvement of CNS involvement, at least two noncontiguous sites [17] or cryptococcemia [17]. Local cryptococcosis [17] is defined as pulmonary cryptococcosis with or without pleural effusion or as focal lymphadenopathy. Our study showed that the proportions of disseminated disease in the AIDS, OTR, NHNT-1, and NHNT-2 groups were 86.7%, 80%, 50%, and 11.5%, respectively, and the difference between groups reached statistical significance (*P* < 0.001) (Table 1). It is noteworthy that the dissemination rate in the NHNT-1 group was much higher than we had expected. One reason may be that most of these patients had been receiving aggressive treatment with immunosuppressants before the onset of cryptococcosis.

### T-lymphocyte subgroups

**Fig. 1:**
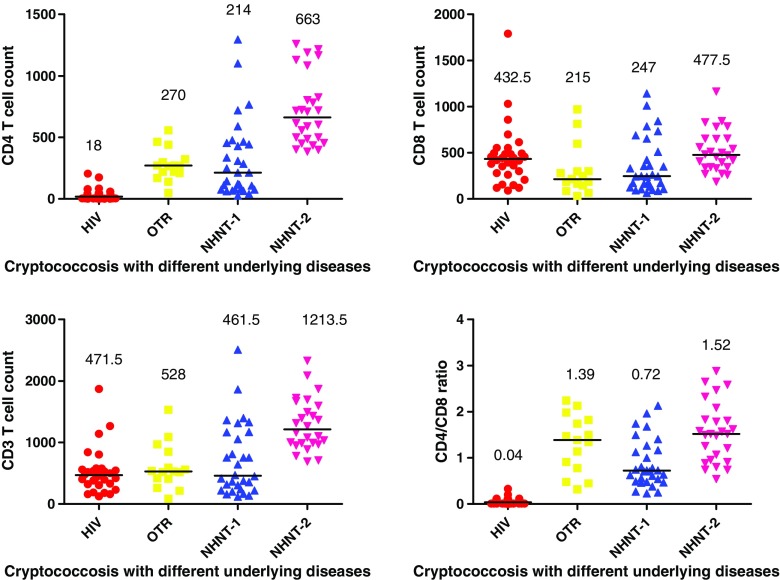
**T**-lymphocyte counts of 101 patients with cryptococcosis, 2009–2015; (**a**) CD4^+^, (**b**) CD8^+^, (**c**) CD3^+^, and (**d**) CD4^+^/CD8^+^ ratio. The median was used to describe the data. The Kruskal-Wallis H test was applied in comparing the four groups; subsequently, the Nemenyi test was used in paired comparisons

The median of CD4^+^ T cell counts in the AIDS group was much lower than that in the OTR, NHNT-1, or NHNT-2 groups, with statistical significance (median, 18 vs 270, 214, and 663; *P* < .001). The median of CD4^+^ T-cell counts in the NHNT-2 group was greater than that in the OTR group (median, 663 vs 270; *P* < .05) and NHNT-1 group (median 663 vs 214; *P* < .005) (Fig. 1a).

CD8^+^ T-cell counts in the OTR and NHNT-1 groups were lower than those in the AIDS and NHNT-2 groups (median, 215, 247 vs 432.5, 477.5; *P* = 0.004) (Fig. 1b). The median of CD3^+^ T-cell counts in the NHNT-2 group was much higher than that in the AIDS, OTR, and NHNT-1 groups, with statistical significance (1213.5 vs 471.5, 528, 461.5; *P* < 0.001) (Fig. 1c).

CD4^+^/CD8^+^ ratio in the AIDS group was lower than OTR, NHNT-1 and NHNT-2 groups with statistical significance (median, 0.04 vs 1.39, 0.72, 1.52; *P* < 0.001). The difference in CD4^+^/CD8^+^ ratio between the NHNT-1 group and the NHNT-2 group reached statistical significance (median, 0.72 vs 1.52; *P* < 0.05) (Fig. 1d).

### The predictive value of these parameters in assessing the risk of disseminated cryptococcosis

First, our study sought to determine whether the parameters of cell-mediated immunity are more accurate than the underlying disease burden in predicting the incidence of dissemination. Second, we sought to discover which of the T-lymphocyte subgroups would be the most sensitive predictor. We applied the ROC curves and Youden’s index to determine the cutoff value of T-lymphocyte subgroups. Then we found the cutoff value of CD4^+^ T cell counts of 400/uL for predicting dissemination; the sensitivity and specificity were 80% and 98.2%. The area under concentration-time curve (AUC) was 0.903 and the 95% confidence interval ranged from 0.835 to 0.970 (These data are shown in ESM_Figure2A). Similarly, supposing 617/μL to be the cutoff value of CD3^+^ T-cell counts, the sensitivity and specificity to predict the dissemination were 86.7% and 82.1%, respectively. The AUC was 0.890 and the 95% confidence interval ranged from 0.823 to 0.957 (the data are shown in ESM_Figure2B). Because of the poor result of AUC in CD8^+^ T cells (0.732) and the CD4^+^/CD8^+^ ratio (0.777), the normal lower limit (242/μL, 0.90) was chosen as the cutoff value.

Then we reclassified the patients according to the following criteria: (1) Underlying disease: the patients with AIDS/OTR (n = 45) were classified in the group with severe underlying disease (group A) and the remainders (n = 56) in the group without severe underlying disease (group B). (2) CD4^+^/CD8^+^ ratio: the patients with a CD4^+^/CD8^+^ ratio below the normal lower limit (n = 59) were classified in group C, and the others (n =42) in group D. (3) CD8^+^ T-lymphocyte count: the patients with CD8^+^ T-lymphocyte counts lower than 242/μL (n = 28) were classified in group E and the others (n = 73) in group F. (4) CD3^+^ T-lymphocyte count: the patients with CD3^+^ T-lymphocyte counts below 617/μL (n = 52) were classified in group G and the remaining patients (n = 49) in group H. (5) CD4^+^ T-lymphocyte count: the patients with CD4^+^ T-lymphocyte counts below 400/μL (n = 63) made up group I, while the normal (n = 38) patients were classified in group J. The differences between groups in terms of most baseline characteristics according to different classification criteria were comparable (*P* > 0.05) (these data are shown in ESM_Table).

On further study, we evaluated the predictive role of underlying diseases and T-cell subgroups regarding disease severity and prognosis according to the various classifications. First, we screened the risk factors that could best predict the general distribution of lesions by single-factor logistic regression analysis. Our study showed that both underlying disease and the parameter of T-lymphocyte subgroups had an effective role in predicting the risk of disseminated cryptococcosis. However, we did not find a close link between male gender/age and incidence of dissemination (Table 2). In consideration of interactions, multivariable logistic regression analysis was applied, and our data suggested that the CD4^+^ T-lymphocyte count was the most sensitive predictor; the odds ratio was 23.3 (95% CI: 2.6–209.7; *P* = .005), while the other parameter did not reach statistical significance (*P* > 0.05) (Table 3). With regard to influence on the distribution of pulmonary lesions, the proportion of unilateral lung lesion in groups A, C, E, G, and I were 66.7%, 61.5%, 42.9%, 53.6%, and 55.6% vs 44.1%, 58.6%, 64.8%, 65%, and 65.6% in groups B, D, F, group H, and J, respectively. There was no statistical significance in these comparisons (*P* > 0.05) (Table 4).

**Table 2:** The results of predictors in assessing the risk of disseminated lesions among 101 patients with cryptococcosis, 2009–2015

Characteristics	Disseminated, n = 56	Localized, n = 45	Odds ratio (95% CI)	*P* valve
Male (n = 73)	38 (67.9%)	35 (77.8%)	0.6(0.2–1.5)	0.270
Age < 50 (n = 78)	47 (83.9%)	31 (68.9%)	2.4(0.9–6.1)	0.077
Severe underlying disease (n = 45)	38 (67.9%)	7 (15.6%)	11.5(4.3–30.6)	<0.001
Decreased CD4^+^/CD8^+^ ratio (n = 59)	43 (76.8%)	16 (35.6%)	6.0(2.5–14.3)	<0.001
Decreased CD8^+^ T-cell count (n = 28)	25 (44.6%)	3 (6.7%)	11.3(3.1–40.8)	<0.001
Decreased CD3^+^ T-cell count (n = 52)	46 (82.1%)	6 (13.3%)	29.9(10.0–89.7)	<0.001
Decreased CD4^+^ T-cell count (n = 63)	54 (96.4%)	9 (20%)	108.0(22.0–529.2)	<0.001

Single-factor logistic regression analysis was used to screen the predictors in assessing the risk of dissemination cryptococcosis

**Table 3:** The results of predictors in assessing the risk of disseminated lesion among 101 patients with cryptococcosis, 2009–2015

Characteristic	Disseminated, n = 56	Localized, n = 45	Odds ratio (95% CI)	*P* valve
Severe underlying disease (n = 45)	38 (67.9%)	7 (15.6%)	2.9(0.7–13.1)	0.161
Decreased CD4^+^/CD8^+^ ratio (n = 59)	43 (76.8%)	16 (35.6%)	1.3(0.2–8.1)	0.775
Decreased CD8^+^ T cell count (n = 28)	25 (44.6%)	3 (6.7%)	3.0(0.4–21.9)	0.280
Decreased CD3^+^ T-cell count (n = 52)	46 (82.1%)	6 (13.3%)	2.2(0.4–12.4)	0.357
Decreased CD4^+^ T-cell count (n = 63)	54 (96.4%)	9 (20%)	23.3(2.6–209.7)	0.005

Multivariable logistic regression analysis was applied in predicting the risk of disseminated cryptococcosis

**Table 4:** Classification standards and distribution of pulmonary lesions

Characteristic	Unilateral lung, n = 41	Bilateral lung, n = 27	*P* value
Underlying disease			
Group A (n = 24)	16(66.7%)	8(33.3%)	0.428
Group B (n = 44)	25(44.1%)	19(55.9%)	
CD4^+^/CD8^+^ ratio			
Group C (n = 39)	24(61.5%)	15(38.5%)	0.808
Group D (n = 29)	17(58.6%)	12(41.4%)	
CD8^+^ T cell count			
Group E (n = 14)	6(42.9%)	8(57.1%)	0.135
Group F (n = 54)	35(64.8%)	19(35.2%)	
CD3^+^ T cell count			
Group G (n = 28)	15(53.6%)	13(46.4%)	0.343
Group H (n = 40)	26(65%)	14(35%)	
CD4^+^ T cell count			
Group I (n = 36)	20(55.6%)	16(44.4%)	0.397
Group J (n = 32)	21(65.6%)	11(34.4%)	

Chi-square test was used to compare the difference in proportion of bilateral lung lesion between two groups

### The role in assessing the disease severity and mortality

A moderate negative correlation was found between the CD4^+^ T-cell count and Apache II score. The correlation coefficient was −0.609 (*P* < 0.001), which was higher than that of CD3^+^ T-cell count (−0.572), underlying disease (−0.471), CD8^+^ T-cell count (−0.384) and CD4^+^/CD8^+^ ratio, respectively (−0.276) (*P* < 0.05) (Table 5). Our study further evaluated the predictive role of underlying disease and T-lymphocyte subgroups with regard to mortality. The mortality of the patients with decreased CD4^+^ T lymphocytes was 23.8%, which was higher than that (5.3%) of the patients with normal CD4^+^ T lymphocyte (*P* = 0.016). The mortality of cryptococcosis patients with severe underlying diseases was slightly higher than that in the patients with relatively mild diseases, but the difference did not reach statistical significance (24.4% vs 10.7%; *P* = 0.067). There was also no statistical significance among the other parameters (*P* > 0.05) (Table 6).

**Table 5:** Relation between the classification standards and the scores of APACHE II

Characteristic	APACHE II score			Correlation coefficient	*P* value
	≤5	6 ∼ 10	≥11		
Underlying disease					
Group A (n = 45)	6	16	23	−0.471	<0.001
Group B (n = 56)	38	5	13		
CD4^+^/CD8^+^ ratio					
Group C (n = 59)	16	20	23	−0.276	0.006
Group D (n = 42)	28	1	13		
CD8^+^ T cell count					
Group E (n = 28)	3	9	16	−0.384	<0.001
Group F (n = 73)	41	12	20		
CD3^+^ T cell count					
Group G (n = 52)	6	19	27	−0.572	<0.001
Group H (n = 49)	38	2	9		
CD4^+^ T-cell count					
Group I (n = 63)	11	20	32	−0.609	<0.001
Group J (n = 38)	33	1	4		

The Spearman correlation analysis was used in comparison

**Table 6:** Mortality and classification standards

Characteristic	Death^a^, n = 17	Survival, n = 84	*P* value
Underlying disease			
Group A (n = 45)	11(24.4%)	34(75.6%)	0.067
Group B (n = 56)	6(10.7%)	50(89.3%)	
CD4^+^/CD8^+^ ratio			
Group C (n = 59)	12(20.3%)	47(79.7%)	0.264
Group D (n = 42)	5(11.9%)	37(88.1%)	
CD8^+^ T-cell count			
Group E (n = 28)	6(21.4%)	22(78.6%)	0.640
Group F (n = 73)	11(15.1%)	62(84.9%)	
CD3^+^ T-cell count			
Group G (n = 52)	10(19.2%)	42(80.8%)	0.507
Group H (n = 49)	7(14.3%)	42(85.7%)	
CD4^+^ T-cell count			
Group I (n = 63)	15(23.8%)	48(76.2%)	0.016
Group J (n = 38)	2(5.3%)	36(94.7%)	

Chi-square test was used to compare the difference in mortality of cryptococcosis between two groups

^a^Attributed death

## Discussion

As far as we know, data on T-lymphocyte subgroups in patients with cryptococcosis have been derived mainly from AIDS patients, whereas studies related to non-AIDS patients have been rare. Therefore our study reviewed the data on T-lymphocyte subgroups in patients with cryptococcosis from eastern China and considered the role of cell immunity in assessing disease severity and prognosis.

We observed consistent results in AIDS patients, whose CD4^+^ T lymphocytes were lower than 50/μL in 83.3% cases and lower than 200/μL in 96.7% cases. Previous research also found CD4^+^ T-lymphocyte counts lower than 50/μL in 78% and 200/μL in 97% of AIDS patients suffering from cryptococcosis [23]. Inversion of the CD4^+^/CD8^+^ ratio was found in all the AIDS patients in our study [16]. Furthermore, a clear-cut result was derived from the NHNT-2 patients in that most of their CD4^+^ and CD8^+^ T-cell counts were normal, but the CD4^+^/CD8^+^ ratio was decreased in some patients.

Other than AIDS and NHNT-2 patients, a heterogeneous result was obtained in OTR and NHNT-1 patients. The number of CD4^+^ T-lymphocytes decreased in 80% and CD8^+^ T cells decreased in 53.3% of the OTR patients, but their CD4^+^/CD8^+^ ratios generally remained normal. Some patients in the NHNT-1 group resembled those in the AIDS or OTR groups and others were similar to patients in the NHNT-2 group. Overall we found that cryptococcosis patients exhibit an obvious heterogeneity of immune status.

The levels of CD8^+^ T cells were normal or increased in the AIDS patients, whereas they were sharply decreased in the OTR patients and those receiving large doses of immunosuppressants. It should be emphasized that a decrease in the CD8^+^ T-cell count can lead to a normal CD4^+^/CD8^+^ ratio in patients with low numbers of CD4^+^ lymphocytes [24]. In short, our study suggests that the value of CD4^+^/CD8^+^ ratios, CD8^+^ T-cell counts, or CD3^+^ T-cell counts is relatively limited in determining the immune status of patients with cryptococcosis. In a word, the CD4^+^ T-cell count was felt to be the most valuable parameter in evaluating the cell immunity of the patients in our study.

Disseminated cryptococcosis was tightly linked with the immune status of cryptococcosis patients. Granuloma was among the most common pathological result of pulmonary lesions in cryptococcosis patients; this was mainly induced by CD4^+^ T-cell immunity [25]. Integrated granuloma may prevent the dissemination of cryptococcal disease. Recent studies show that Th1/Th17 lymphocytes, which are CD4^+^ T-cell subgroups, may prevent dissemination [26]. The CD4^+^ T-cell count was sharply decreased (median, 82/μL; range 7–292/μL) in 53 patients with idiopathic CD4^+^ T lymphocytopenia and cryptococcosis [19]. There was CNS involvement in 75.4% of these patients [19]. CD4^+^ T-cell counts were also decreased in eight patients with systemic lupus erythematosus who also suffered from cryptococcal meningitis (mean ± SD, 113.2 ± 59.2/μL) [27]. One AIDS study revealed that CD4^+^ T-cell counts in patients with local cryptococcosis were higher than in those with disseminated cryptococcosis [28]. Our study confirmed that as opposed to underlying disease, the CD4^+^ T-cell count is a more effective predictor of disseminated cryptococcosis.

Recent studies have reported that the proportions of patchy shadow, consolidation, and cavities in immunocompromised patients were higher than in immunocompetent patients [21, 29, 30]; therefore, we speculated that imaging of the chest in our immunocompromised patients might be more severe than it was in those who were immunocompetent. However, we found that neither underlying disease nor T-lymphocyte subgroups were good predictors for the distribution of lung lesions. Since inhaled cryptococcal spores are the main source of infection, we assumed that their presence might be more important as an indicator of lung lesions.

Joseph et al. discovered that the Cryptococcus-specific CD4^+^ memory T-cell response to *Cryptococcus* is closely correlated with clinical severity and outcome in AIDS patients with cryptococcal meningitis [31]. Our study suggests that the total CD4^+^ T-cell count is also effective in assessing disease severity and mortality among patients either with or without HIV infection.

Inevitably, our study has some limitations. First, it is a retrospective study. Second, our study retrieved data from of T-lymphocyte subgroups at only one time point and before antifungal therapy. Dynamic changes in T-lymphocyte subgroups during the follow-up period were not clear. In a further study, therefore, a prospective design and dynamic follow-up of T-lymphocyte subgroups will be needed.

In conclusion, the CD4^+^ T-cell count is an effective indicator of a patient’s immune status and provides a more accurate estimate of disease severity and prognosis in cases of cryptococcal infection. In other words, a count of CD4+ T cells in the peripheral blood of each patient with cryptococcosis should be done routinely. For those with low CD4^+^ T-cell counts, testing for possible dissemination should proceed as promptly as possible.

## Electronic supplementary material

Below is the link to the electronic supplementary material.

ESM 1 (DOCX 31 kb)

ESM 2 (DOCX 13 kb)
