# Supplementary material for: The CD4+ T-lymphocyte count is an important predictor for the prognosis of cryptococcosis
Source: Eur J Clin Microbiol Infect Dis. 2016 Dec 29;36(5):897–904. doi: 10.1007/s10096-016-2880-9 (PMC5395594; doi:10.1007/s10096-016-2880-9)
Supplement: Supplementary file 1 — (DOCX 31 kb) [file 10096_2016_2880_MOESM1_ESM.docx]

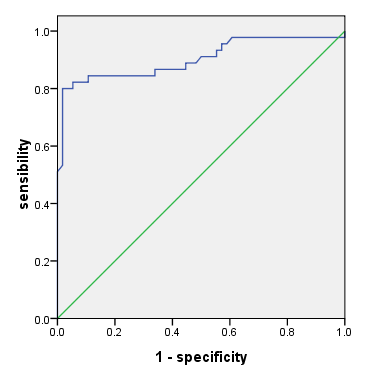


**ESM_Figure2A. Receiver operating characteristics curve of CD4^+^ T-lymphocyte count.**


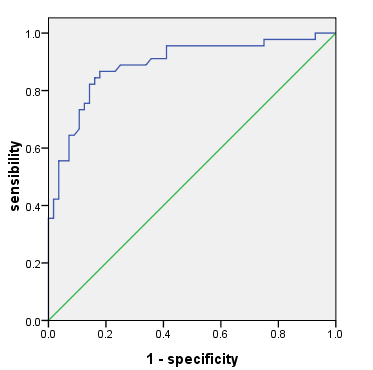


**ESM_Figure2B. Receiver operating characteristics curve of CD3^+^ T-lymphocyte count.**
