# Supplementary material for: The CD4+ T-lymphocyte count is an important predictor for the prognosis of cryptococcosis
Source: Eur J Clin Microbiol Infect Dis. 2016 Dec 29;36(5):897–904. doi: 10.1007/s10096-016-2880-9 (PMC5395594; doi:10.1007/s10096-016-2880-9)
Supplement: Supplementary file 2 — (DOCX 13 kb) [file 10096_2016_2880_MOESM2_ESM.docx]

**Patients’ baseline characteristics according to 5 different classification standards**

|  | Male | Age | *P* value |
| --- | --- | --- | --- |
| **Underlying disease** |  |  |  |
| Group A (n=45) | 37 (82.2%) | 39.5±12.1 | ^*^.045 |
| Group B (n=56) | 36 (64.3%) | 43.1±14.4 | ^†^.186 |
| **CD4^+^/CD8^+^ ratio** |  |  |  |
| Group C (n=59) | 43 (72.9%) | 40.3±15.0 | ^*^.872 |
| Group D (n=42) | 30 (71.4%) | 43.1±11.0 | ^†^.291 |
| **CD8^+^ T-cell count** |  |  |  |
| Group E (n=28) | 17 (60.7%) | 40.1±14.9 | ^*^.108 |
| Group F (n=73) | 56 (76.7%) | 42.0±13.0 | ^†^0.526 |
| **CD3^+^ T-cell count** |  |  |  |
| Group G (n=52) | 35 (67.3%) | 38.9±13.5 | ^*^.250 |
| Group H (n=49) | 38 (77.6%) | 44.2±13.1 | ^†^.050 |
| **CD4^+^ T-cell count** |  |  |  |
| Group I (n=63) | 44 (69.8%) | 39.4±13.7 | ^*^.481 |
| Group J (n=38) | 29 (76.3%) | 44.8±12.6 | ^†^.051 |

^*^This figure is the *P* value for gender. ^†^This figure is the *P* value for age.

Two-independent sample T-tests and Chi-square tests were applied in comparison.
